# Comparison of Electrophoretic and Bromocresol Green Albumin Methods in Chickens and Other Veterinary Species

**DOI:** 10.1111/vcp.70015

**Published:** 2025-06-02

**Authors:** Jeffrey Brandon, Heather Reider, Kristy L. Pabilonia, A. Russell Moore

**Affiliations:** ^1^ Department of Microbiology, Immunology and Pathology Colorado State University Fort Collins Colorado USA; ^2^ Veterinary Diagnostic Laboratories Colorado State University Fort Collins Colorado USA

**Keywords:** avian, Bland–Altman, cat, dog, horse, protein, regression, ruminant

## Abstract

**Background:**

The bromocresol green albumin assay (ALB_BCG_) has been used in birds and reportedly is noncomparable with electrophoretic albumin (ALB_PE_) in many species. It is accepted for use in some species and rejected in others.

**Objectives:**

We aimed to compare the performance of ALB_PE_ and ALB_BCG_ methods within backyard chickens and compare the performance of ALB_BCG_ in chickens with other veterinary species where the ALB_BCG_ method is accepted and used clinically.

**Methods:**

Chicken plasma collected during reference interval development and samples submitted for diagnostic biochemistry profile were evaluated using the ALB_BCG_ and ALB_PE_ assays. Method comparison was performed according to current recommendations, including the use of Passing–Bablok and Bland–Altman analysis. ALB_BCG_ and ALB_PE_ were also measured in other avian species, dogs, cats, horses, and domestic ruminants. Method comparison was evaluated within and between species, including clinical utility based on the percentage of cases discordantly interpreted as hypo‐, normo‐, or hyperalbuminemic by ALB_BCG_ and ALB_PE_.

**Results:**

In chickens, ALB_BCG_ and ALB_PE_ were not comparable, having a constant bias (−0.4 g/dL) and proportional bias. Similarly, the methods were not comparable in other species; > 10% of samples had > TE_A_ (15%) difference in all species. The clinical utility of albumin interpretation in chickens did not differ significantly from that in dogs and horses, as determined by ANOVA.

**Conclusions:**

The data suggest that ALB_BCG_ is not comparable with ALB_PE_ and performs similarly across all tested species. There is no evidence to support the continued rejection of the ALB_BCG_ in chicken and other avians and acceptance in some mammals.

## 1 Introduction

Increasing numbers of small privately owned backyard chicken (*Gallus gallus domesticus*) flocks are being kept and viewed as both pets and production animals [1]. This has resulted in an increased need for backyard poultry diagnostic medicine to include more antemortem diagnostic evaluations, including the assessment of plasma proteins.

Protein electrophoresis has long been held as the reference standard for assessment of protein fractions, including albumin (ALB_PE_) in veterinary medicine [2, 3, 4, 5, 6]. Bromocresol green (BCG) based albumin methods are commonly used in veterinary reference laboratories and clinics, though they have been shown to be non‐comparable with ALB_PE_ in avians [6, 7, 8, 9, 10]. Despite this non‐comparability, many publications report the use of mammalian‐targeted BCG albumin (ALB_BCG_) assays in avian diagnostic cases as a screening method for plasma albumin concentration [2, 9, 11, 12, 13, 14, 15]. Additionally, there are reports of ALB_BCG_ being used as a reference standard for evaluating the performance of point‐of‐care analyzers in birds [11, 13, 14, 15]. Non‐comparability between ALB_PE_ and ALB_BCG_ has been reported in several mammalian species, where the BCG method is commonly used in routine biochemical panels to estimate albumin [16, 17, 18, 19, 20]. Based on our experience, the degree of non‐comparability between ALB_PE_ and ALB_BCG_ assessments and the frequency of notably inaccurate ALB_BCG_ results are similar between avians and mammals.

Therefore, the objectives of this study were to compare the performance of ALB_PE_ and ALB_BCG_ methods within backyard chickens and compare the performance of ALB_BCG_ in chickens with other veterinary species where the ALB_BCG_ method is accepted and used clinically.

## 2 Methods

This study was approved by the Institutional Animal Care and Use Committee at Colorado State University.

### 2.1 Samples

Samples used for heparin plasma protein electrophoresis RI generation in backyard chickens in northern Colorado, published elsewhere, were used in this study. Additionally, heparin plasma samples from chickens and other non‐chicken avian species (Avian_Other_) were included, as well as serum samples from dogs, cats, horses, and domestic ruminants (sheep, goats, cows) submitted to the CSU Clinical Pathology Laboratory for routine biochemistry evaluation. All samples lacked significant lipemia (lipemia index < 150) or hemolysis (hemolysis index < 500). The collection targeted a minimum of 30 random samples from each group [21]. To allow comparison of ALB_BCG_ method performance over the same analytical range, samples with an ALB_BCG_ ≤ 2.5 g/dL were included as a targeted collection from dogs, cats, horses, and ruminants.

### 2.2 Albumin Measurement

All samples had a biuret total protein (TP), ALB_BCG_, colorimetric globulin (Glob) calculated as the difference between TP and ALB_BCG_, Alb_BCG_:Glob (A:G_BCG_), and protein electrophoresis performed. The TP and ALB_BCG_ were performed on a commercial biochemistry analyzer using commercially available reagents (Cobas c501; Roche Diagnostics) at the time of submission. Samples were stored at −20°C and electrophoresis was performed in batches without multiple freeze–thaw cycles of samples. Protein electrophoresis was conducted using non‐reduced amido black stained agarose gel electrophoresis (Sebia Hydrasys with Hydragel Protein (E) with amido black kit, Sebia Inc., Norcross, GA, USA). All electrophoretic evaluations were completed within 3 months of sample collection. Protein fraction demarcations were made relative to published data [22, 23, 24, 25].

### 2.3 Analytical Performance Characterization

All albumin methods used in this study operated within expected quality parameters as defined in the laboratory for mammalian samples. For the ALB_BCG_ assays, the laboratory's routine commercial quality control materials (QCM, Bio‐Rad Multiqual 1 and Multiqual 3; Bio Rad, Hercules, CA, USA) were used. Adequate performance of ALB_BCG_ in chickens was verified using duplicate measurements of 15 chicken plasma samples across runs, following the within‐subject standard deviation method [26]. The performance of ALB_PE_ was evaluated per the laboratory's protocol using pooled dog and cat serum as QCM and assessing interrun CV_%_; adequate performance was verified in chickens using 5 interrun repeat measurements of a single chicken plasma sample to calculate CV_%_.

The linearity of ALB_BCG_ in chicken plasma was evaluated using archived chicken plasma in two studies. A dilutional linearity study was completed by diluting a sample with phosphate‐buffered saline to produce seven samples with a range of ALB_BCG_ from 0.26 to 2.5 g/dL and a constant electrophoretic A:G (A:G_PE_ 0.88). A mixing linearity study was completed by mixing samples with high and low A:G_BCG_ to produce seven samples with a range of A:G_BCG_ from 0.14 to 0.87 and ALB_BCG_ from 1.3 to 2.5 g/dL. All linearity samples were evaluated for TP and ALB_BCG_ in duplicate, and current guidelines for evaluation of linearity were followed [27].

### 2.4 Method Comparison

Spearman's rank correlation coefficient (*ρ*) was used to evaluate the correlation of the albumin methods. Method comparison included Passing–Bablok (P–B) regression and Bland–Altman (B–A) difference plot analysis, including the calculation of the 95% limits of agreement, following current ASVCP guidelines [27]. To assess the clinical relevance of using the methods interchangeably, limits of agreement based on TE_A_ (15%) were also used for B‐A analysis, and the percentage of samples outside these limits was tabulated [28, 29]. The samples for each species were stratified based on ALB_BCG_ (≤ 2.5 g/dL vs. > 2.5 g/dL) and method comparison was performed on each of the stratified species groups. The correlation of the methods in chickens was compared with the correlation in other species at the expected chicken range (≤ 2.5 g/dL) and the normal range for the species (i.e., > 2.5 g/dL for mammals) using Fisher's *Z*‐transformation [30]. The magnitude of the difference between methods was compared between species using an ANOVA with Dunnett's multiple comparison on the absolute value of the difference (Diff_ABS_) between the methods.

Because essentially all species had an unacceptably high frequency of samples with > TE_A_ difference, the agreement of interpretation based on method‐specific RI was also evaluated. For this, RIs were calculated for ALB_BCG_, Glob, and the A:G_BCG_ from the samples used for chicken electrophoretic RIs according to current ASVCP guidelines and as outlined for the chicken ALB_PE_ RI [31]. Laboratory‐accepted RIs for ALB_BCG_ and A:G_BCG_ were available for all mammals. Laboratory‐accepted RIs for electrophoretic evaluation were available for dog and cat samples. Published electrophoretic RIs for horses and ruminants were used [24, 25]. The RIs derived for chickens were used to evaluate data from Avian_other_ for all measurands. Samples used for RI development were excluded from the dataset, and the ALB_BCG_ and ALB_PE_ results were classified as hypoalbuminemic, normoalbuminemic, or hyperalbuminemic based on the species and method‐specific RIs. The frequency of samples with discordant interpretation between the ALB_BCG_ and ALB_PE_ was compared using Fisher's exact test.

Statistical analyses were completed using Microsoft Excel (Microsoft Office 2016; Microsoft) and the Reference Value Advisor macro, GraphPad Prism 9 (GraphPad Software Inc.), and MedCalc (v 19.7; MedCalc Software Ltd) [32]. Alpha was set at 0.05 unless otherwise stated.

## 3 Results

### 3.1 Samples

The number and protein characteristics of the samples used in this study are presented in Table 1. The Avian_Other_ group included seven Red Tailed Hawks, four Swainson's Hawks, four Eastern Screech Owls, three Cinerous Vultures, two each of American Kestrel, Great Horned Owl, and domestic duck, and a single representative of 14 other species.

**TABLE 1:** Comparison of total protein, electrophoretic albumin (ALB_PE_) and electrophoretic A:G (A:G_PE_) between species groups. The data was stratified based on the BCG albumin concentration (ALB_BCG_).

	All							ALB_BCG_ < 2.5							Alb_BCG_ > 2.5						
	*n*	Total protein		Alb_PE_		A:G_PE_		*n*	Total protein		Alb_PE_		A:G_PE_		*n*	Total protein		Alb_PE_		A:G_PE_	
		Mean (g/dL)	SD (g/dL)	Mean (g/dL)	SD (g/dL)	Mean	SD		Mean (g/dL)	SD (g/dL)	Mean (g/dL)	SD (g/dL)	Mean	SD		Mean (g/dL)	SD (g/dL)	Mean (g/dL)	SD (g/dL)	Mean	SD
Chicken	162	5.29	0.91	2.1	0.42	0.73	0.25	162	5.29	0.91	2.1	0.42	0.73	0.25	0						
Avian_Other_	37	3.38	0.85	1.35	0.44	0.72	0.3	37	3.38	0.85	1.35	0.44	0.72	0.3	0						
Dog	142	5.45	1.22	2.49	0.84	0.89	0.32	68	4.66	1.31	1.69	0.39	0.65	0.23	74	6.18	0.41	3.23	0.3	1.12	0.2
Horse	106	5.9	1.52	2.36	0.71	0.79	0.32	74	5.59	1.7	1.99	0.49	0.71	0.33	32	6.63	0.53	3.19	0.34	0.98	0.22
Cat	64	6.56	1.53	2.85	0.94	0.85	0.39	32	5.85	1.78	2.03	0.36	0.64	0.34	32	7.27	0.69	3.66	0.55	1.07	0.31
Ruminant	76	6.61	1.42	2.88	0.8	0.85	0.33	45	6.23	1.58	2.39	0.5	0.72	0.33	31	7.17	7.17	3.59	0.58	1.04	0.25

For samples with an ALB_BCG_ ≤ 2.5 g/dL, one‐way ANOVA with Dunnett's multiple comparison test identified that, when compared to chickens, Avian_Other_ had a lower TP (mean difference 1.91 g/dL, *P* < 0.001) and Alb_PE_ (mean difference 0.57 g/dL, *P* < 0.001); dogs had a lower TP (mean difference 0.63 g/dL, *P* = 0.005) and Alb_PE_ (mean difference 0.06 g/dL, *P* < 0.001); ruminants had a higher TP (mean difference 0.94 g/dL, *P <* 0.001) and Alb_PE_ (mean difference 0.28 g/dL, *P* < 0.001), and there were no detected differences in A:G_PE_ for any group.

### 3.2 Analytical Performance Characterization

**FIGURE 1:**
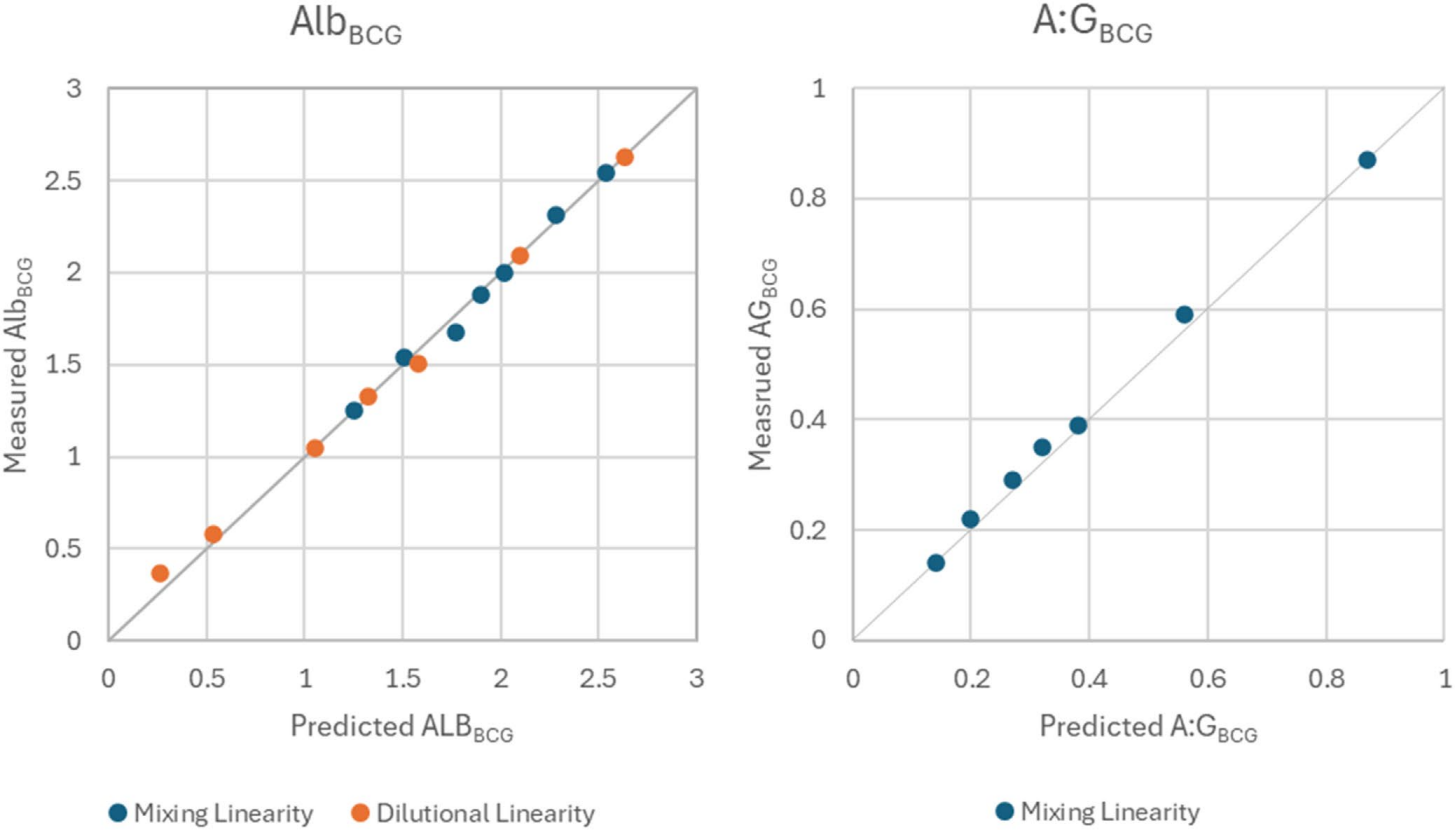
Linearity of colorimetric albumin (ALB_BCG_) and albumin: globulin ratio (A:G_BCG_) evaluated using saline dilution of a single chicken plasma sample or mixing of chicken plasma samples with high and low A:G ratios.

The observed CV_%_ for all tested measurands in chickens was < 5% and was similar to CV_%_ determined using QCM (Table 2). Linearity was documented for ALB_BCG_ in the mixing and dilutional studies, Table 3 and Figure 1. Similarly, A:G_BCG_ measured in the mixing linearity study was linear, as shown in Figure 1. The assays were determined to be performing sufficiently for a single measurement of each sample.

**TABLE 2:** Observed analytical performance for albumin measurands in chickens, dogs, cats and commercial quality control material, Multiqual 1 (QCM1), and Multiqual 3 (QCM3). Data are presented as mean, standard deviation (SD) and interassay coefficient of variation (CV%) when the bromocresol green (ALB_BCG_) and electrophoretic (ALB_PE_) methods were used.

Measurand	Material	Mean (g/dL)	SD (g/dL)	CV%
ALB_BCG_	Chicken	1.72	0.28	3.1
	QCM1	2.74	0.07	2.6
	QCM3	4.19	0.08	1.9
Alb_PE_	Chicken	1.722	0.038	2.2
	Dog	3.487	0.089	2.6
	Cat	4.088	0.031	0.8

**TABLE 3:** Results of linearity studies completed using chicken plasma and the bromocresol green method to measure albumin (ALB_BCG_) and calculate the albumin to globulin ratio (A:G_BCG_). Pearson's *r* and simple linear regression slope and intercept, along with their 95% confidence intervals (CIs), are presented.

	Study type	*r*	Slope (95% CI)	Intercept (95% CI)
ALB_BCG_	Dilutional	0.999	0.96 (0.90–1.01)	−0.03 (−0.02 to 0.16)
ALB_BCG_	Mixing	0.996	1.01 (0.91–1.11)	−0.02 (−0.23 to 0.18)
A:G_BCG_	Mixing	0.999	0.99 (0.93–1.05)	0.02 (−0.01 to 0.05)

### 3.3 Method Comparison

The *P*‐value for the Spearman's *ρ* was < 0.001 for all analyses. Spearman's *ρ* ranged from 0.626 (Avian_Other_ and horses when Alb_BCG_ > 2.5 g/dL) to 0.957 (cat), suggesting at least a moderate correlation of the two methods. Comparison of Spearman's *ρ* data between species using Fisher's *Z*‐transformation is presented in Table S1; this did not detect a difference in the correlation of Alb_BCG_ and ALB_PE_ between chickens and any other species when Alb_BCG_ ≤ 2.5 g/dL or between chickens and dogs, horses, or ruminants when Alb_BCG_ > 2.5 g/dL. Chickens had a lower Spearman's ρ than dogs, cats, and horses when all ALB_BCG_ was considered for these species.

**FIGURE 2:**
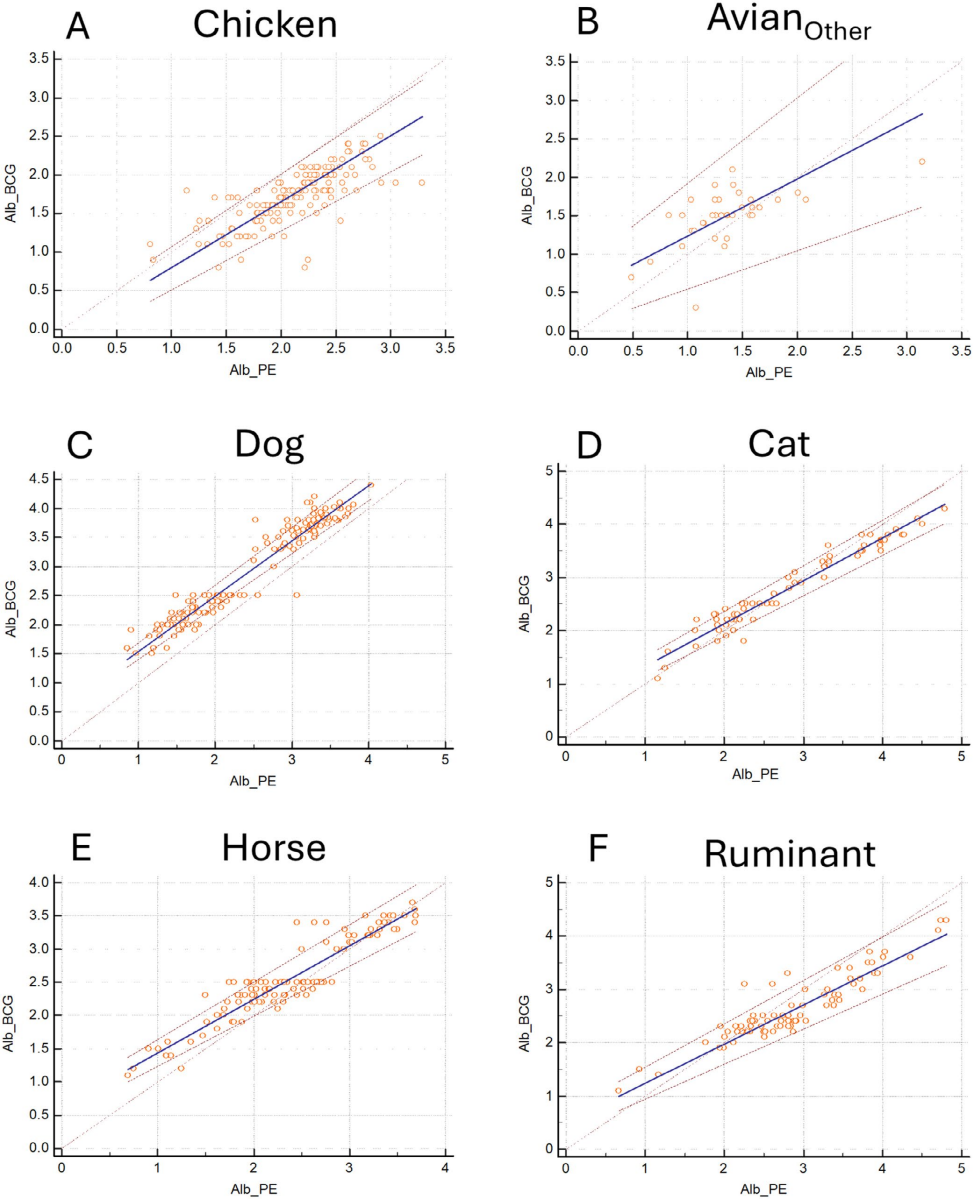
Passing–Bablok regression plots comparing colorimetric albumin (ALB_BCG_) and electrophoretic albumin (ALB_PE_) assessment in various veterinary species.

The results of the P–B regression are presented in Table 4 and Figure 2. The confidence interval of the P–B regression slope or intercept did not include 1 or 0, respectively, for each species. This suggested the presence of constant or proportional bias, or constant and proportional bias between Alb_BCG_ and ALB_PE_ for all species.

**TABLE 4:** Passing–Bablok comparison and Spearman rank correlation (*ρ*) of albumin measured using electrophoresis and colorometric (Alb_BCG_) methods in veterinary species. Data were further stratified based on ALB_BCG_ results. Intercept and slope are presented as observed value (95% CI).

	All				Alb_BCG_ ≤ 2.5				Alb_BCG_ > 2.5			
	*n*	Intercept	Slope	*ρ*	*n*	Intercept	Slope	*ρ*	*n*	Intercept	Slope	*ρ*
Chickens	162	−0.05 (−0.25 to 0.12)	0.85 (0.76–0.94)	0.798	162	−0.05 (−0.25 to 0.12)	0.85 (0.76–0.94)	0.798	0			
Avian_Other_	37	0.49 (0.04 to 0.81)	0.74 (0.5–1.11)	0.626	37	0.49 (0.04 to 0.81)	0.74 (0.5–1.11)	0.626	0			
Dog	142	0.6 (0.49 to 0.71)	0.95 (0.91–0.99)	0.944	68	0.98 (0.73 to 1.15)	0.71 (0.61–0.86)	0.829	74	1.05 (0.58 to 1.48)	0.82 (0.68–0.96)	0.751
Horse	64	0.52 (0.36 to 0.64)	0.81 (0.76–0.86)	0.957	32	0.25 (−0.24 to 0.8)	0.92 (0.67–1.17)	0.720	32	0.89 (0.65 to 1.19)	0.7 (0.63–0.77)	0.937
Cat	106	0.63 (0.49 to 0.77)	0.81 (0.75–0.87)	0.895	74	0.9 (0.73 to 1.13)	0.64 (0.53–0.73)	0.749	32	1.64 (0.92 to 2.2)	0.52 (0.34–0.73)	0.626
Ruminant	76	0.5 (0.29 to 0.73)	0.73 (0.66–0.81)	0.861	45	1.09 (0.83 to 1.43)	0.48 (0.33–0.59)	0.664	31	0.17 (−0.58 to 0.93)	0.85 (0.63–1.04)	0.761

**FIGURE 3:**
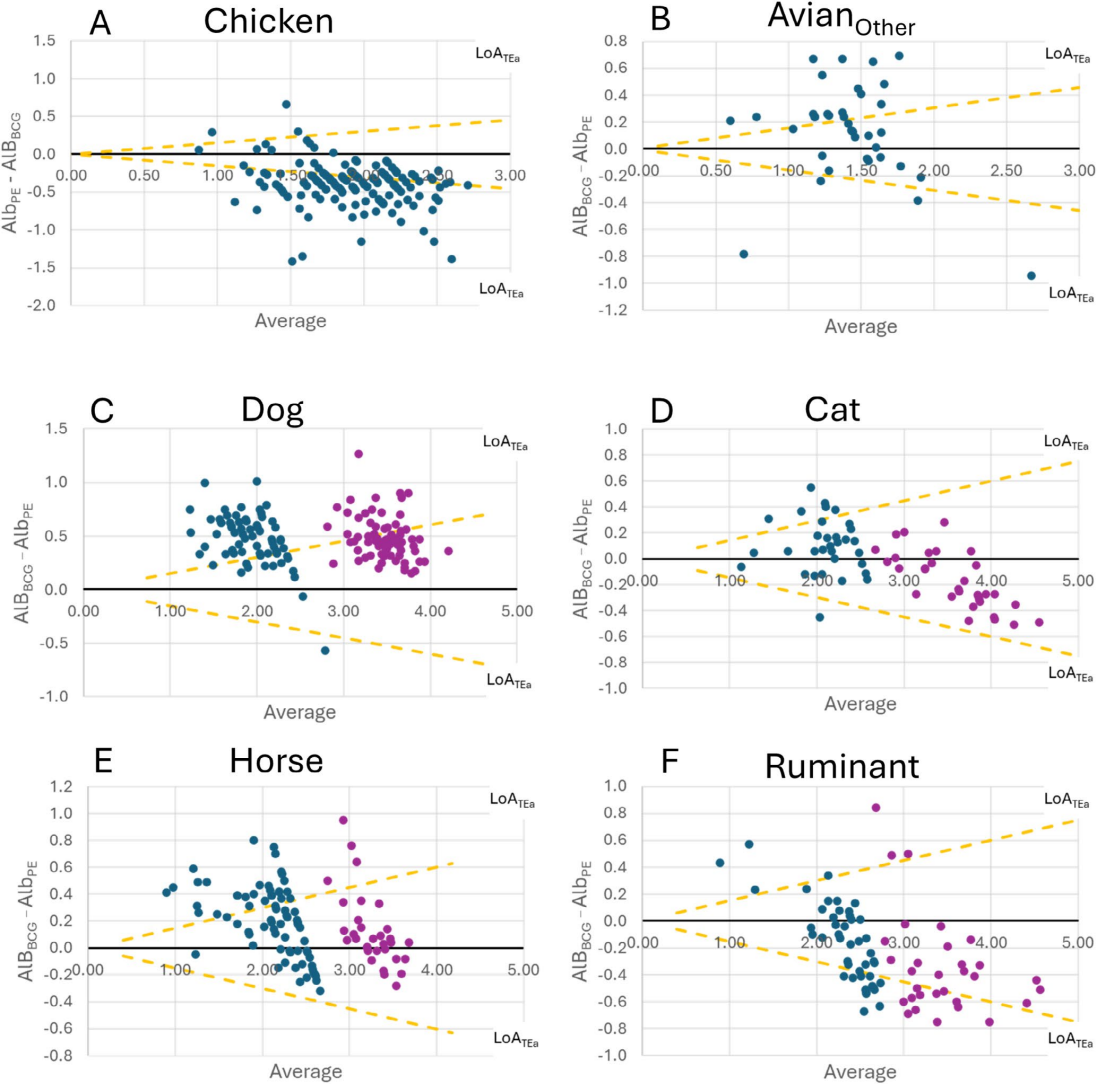
Bland–Altman Difference plots comparing colorimetric albumin (ALB_BCG_) and electrophoretic albumin (ALB_PE_) assessment in various veterinary species. The limits of agreement based on recommended total allowable error (LoA_TEA_) are plotted. The data have been subdivided at ALB_BCG_ ≤ 2.5 g/dL (blue) and ALB_BCG_ > 2.5 g/dL (purple) to allow comparison of a similar analytical range with chickens.

The results of B–A analysis are presented in Table 5 and Figure 3. Close evaluation of the P–B and B–A results highlighted a variable relationship between Alb_BCG_ and ALB_PE_, with Alb_BCG_ being, on average, less than ALB_PE_ for chickens and ruminant samples and greater than ALB_PE_ for horse and dog samples and cat samples with ALB_BCG_ ≤ 2.5 g/dL. Notably, the confidence interval of the mean difference between methods (Diff_Mean_) included zero for the Avian_Other_ and cats when ALB_BCG_ > 2.5 g/dL, indicating that the data were not suggestive of constant bias, though proportional bias or outlier values likely increased the width of the confidence interval and obscured a component of constant bias. More than 10% of samples had a difference greater than TE_A_ (i.e., 15%) between ALB_BCG_ and ALB_PE_ for all evaluated groups, except cats with ALB_BCG_ > 2.5 g/dL. Together, this data were interpreted as evidence that the Alb_BCG_ and ALB_PE_ could not be considered comparable for any of the evaluated species.

**TABLE 5:** Bland–Altman method comparison of albumin assessment using electrophoresis and colorimetric (Alb_BCG_) methods in veterinary species. The data were also stratified based on Alb_BCG_. Data presented are the mean difference (Diff_mean_) with it's 95% confidence interval, 95% limits of agreements (95% LoA), and the percentage outside the TEa LoA or the percentage of samples with greater than 15% difference between methods (> 15%).

	All				Alb_BCG_ < 2.5				Alb_BCG_ > 2.5			
	*n*	Diff_mean_ (g/dL)	95% LoA (g/dL)	> 15% (%)	*n*	Diff_mean_ (g/dL)	95% LoA (g/dL)	> 15% (%)	*n*	Diff_mean_ (g/dL)	95% LoA (g/dL)	> 15% (%)
Chickens	162	−0.4 (−0.44 to −0.36)	−0.95 to 0.15	73.5	162	−0.4 (−0.44 to −0.36)	−0.95 to 0.15	73.5	0			
Avian_Other_	37	0.13 (0.01 to 0.25)	−0.58 to 0.84	59.5	37	0.13 (0.01 to 0.25)	−0.58 to 0.84	59.5	0			
Dog	142	0.47 (0.43 to 0.51)	0.04 to 0.9	57.7	68	0.47 (0.41 to 0.53)	0 to 0.94	83.8	74	0.48 (0.44 to 0.52)	0.09 to 0.87	33.8
Horse	64	−0.03 (−0.09 to 0.03)	−0.52 to 0.46	10.9	32	0.1 (0.02 to 0.18)	−0.31 to 0.51	21.9	32	−0.17 (−0.25 to −0.09)	−0.6 to 0.26	0.0
Cat	106	0.18 (0.12 to 0.24)	−0.35 to 0.71	32.1	74	0.2 (0.14 to 0.26)	−0.31 to 0.71	40.5	32	0.12 (0.02 to 0.22)	−0.43 to 0.67	12.5
Ruminant	76	−0.23 (−0.31 to −0.15)	−0.9 to 0.44	42.1	45	−0.16 (−0.24 to −0.08)	−0.73 to 0.41	37.8	31	−0.34 (−0.48 to −0.2)	−1.07 to 0.39	48.4

**FIGURE 4:**
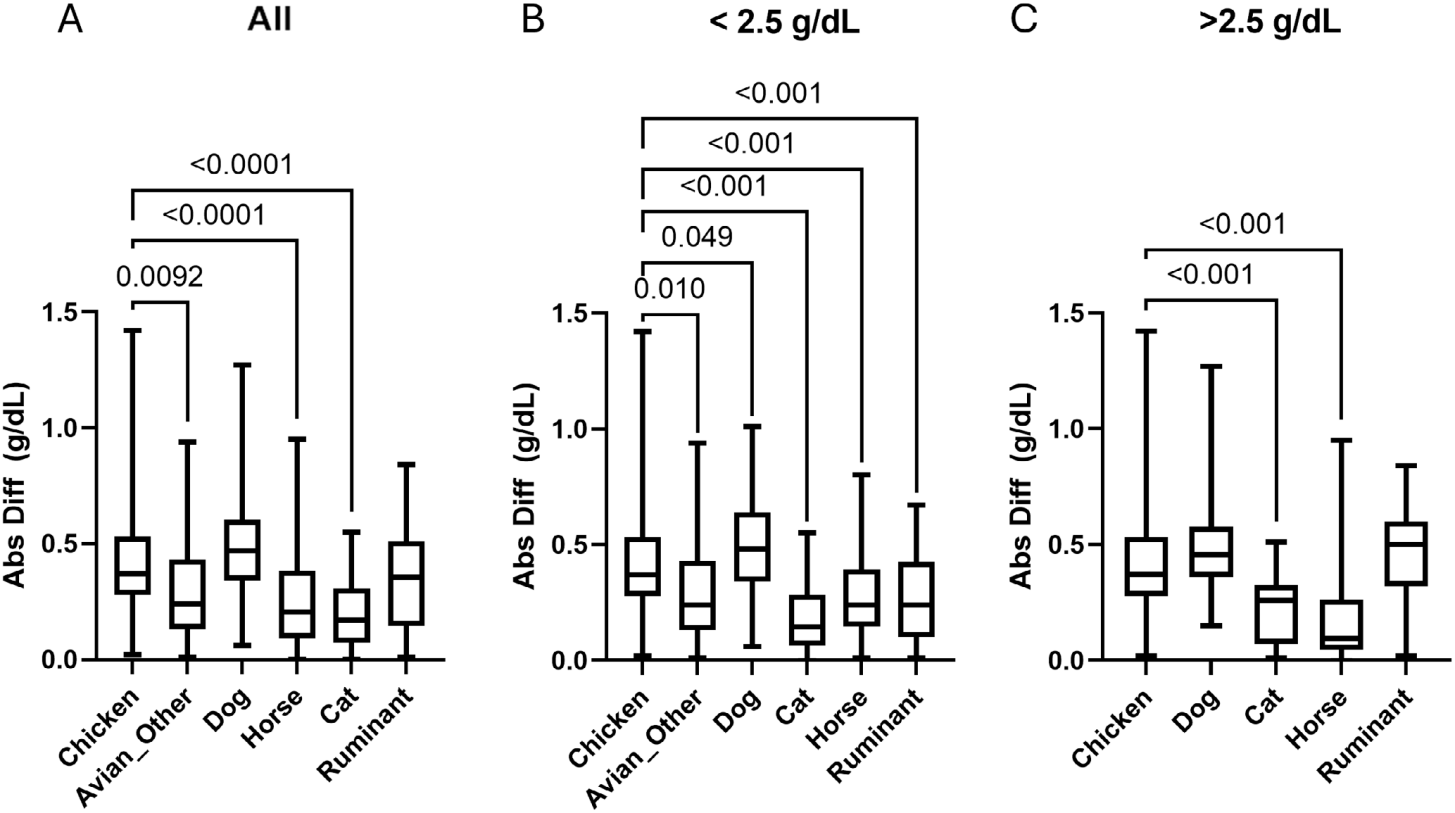
Box plot of the absolute difference (Abs Diff) between colorimetric (Alb_BCG_) and electrophoretic albumin. The Abs Diff observed in chickens was compared to other species when all samples (A), only samples with ALB_BCG_ ≤ 2.5 g/dL (B), or mammalian samples had ALB_BCG_ > 2.5 g/dL (C). The *p*‐value of an ANOVA with Dunnett's multiple comparison is indicated when < 0.05.

Comparison of the Diff_ABS_ between ALB methods for each species is presented in Figure 4. ANOVA with Dunnett's multiple comparison indicated that on average, when all samples were considered, the mean Diff_ABS_ for chicken samples was greater than the mean Diff_ABS_ of Avian_Other_, cats, and horses by 0.12–0.21 g/dL and that there was no difference noted for dogs or ruminants. When ALB_BCG_ ≤ 2.5 g/dL, the chicken mean Diff_ABS_ was greater by 0.12–0.24 g/dL for Avian_Other_, cats, horses, and ruminants, and less than dogs by 0.08 g/dL. When the Diff_ABS_ in chickens was compared with the Diff_ABS_ found in mammals with ABL_BCG_ > 2.5 g/dL, the chicken mean Diff_ABS_ was greater by 0.19 g/dL for cats and 0.23 g/dL for horses, with no difference detected for dogs and ruminants.

Reference Intervals for biochemical protein fractions were calculated from the RI cohort of chickens and partitioned by sex, Table S2. The frequency of discordant interpretations of hypo‐, normo‐, and hyperalbuminemia between ALB_BCG_ and ALB_PE_ is presented in Table 6. When considering the ALB_BCG_ range that included the RI for each species, Fisher's exact test was compatible with a higher frequency of discordant interpretations between Avian_Other_ and ruminants than chickens, while no significant difference in discordant interpretations was found between chickens and horses, cats, or dogs.

**TABLE 6:** Summary of the frequency of discordant interpretations between albumin assessment using electrophoresis and bromocresol green (Alb_BCG_) methods in veterinary species. Discord was defined as disparate interpretation of hyper‐, normo‐, and hypoalbuminemia based on method specific reference intervals. The *P‐* value of a Fisher's exact test against chicken data is provided.

	All				Alb_BCG_ < 2.5				Alb_BCG_ > 2.5			
	*n*	Discord	%	*p*	*n*	Discord	%	*p*	*n*	Discord	%	*p*
Chickens	31	3	9.7		31	3	9.7		0			
Avian_Other_	37	14	37.8	0.011	37	14	37.8	0.011	0			
Dog	142	8	5.6	0.435	68	1	1.5	0.087	74	7	9.5	> 0.999
Cat	64	2	3.1	0.326	32	0	0	0.113	32	2	6.3	0.672
Horse	106	8	7.5	0.712	74	0	0	0.024	32	8	25	0.184
Ruminant	76	23	30.3	0.026	45	6	13.3	0.728	31	17	54.8	0.003

## 4 Discussion

We evaluated the performance of ALB_PE_ and ALB_BCG_ methods in chickens and compared that performance with the performance in other veterinary species where ALB_BCG_ is commonly used. The ALB_BCG_ method was shown to be analytically non‐comparable with ALB_PE_ across all species, with variable performance between species, and a mean difference between methods of 0.1–0.5 g/dL. Furthermore, the clinical performance of ALB_BCG_ for identifying coarse interpretive categories of hypo‐, normo‐, and hyperalbuminemia was less than ideal in all species, with chickens performing similar to, or better than, other species. Overall, the data suggest that ALB_BCG_ provides a coarse assessment of albumin concentration across all evaluated species when ALB_PE_ is considered the reference standard, as recommended in the literature.

The ALB_BCG_ assay for chicken samples met all laboratory quality assurance requirements. This included an acceptable CV_%_ in chickens, which mirrored the assay's performance in mammals. The linearity under dilution with saline suggests that the assay can accurately measure chicken albumin when it is below the RI. The mixing linearity study was performed to estimate the effects of differing A:G ratios on ALB_BCG_ because BCG is known to bind globulins in addition to albumin [18]. The data suggest that decreasing albumin concentration in the face of decreasing A:G (i.e., a higher percentage of globulins) yields a linear series of ALB_BCG_ measurements. It should be considered that albumin and globulin concentrations can vary independently over the course of disease within a single patient. This would yield independent changes in Alb and A:G, which might not produce a linear relationship between ALB_BCG_ and ALB_PE_ in the clinical setting. Nonetheless, ALB_BCG_ appeared to produce repeatable and analytically acceptable results in chickens.

The samples were frozen after assessment of TP and ALB_BCG_ for batch analysis of ALB_PE_ with the longest storage time of 3 months. Freezer stability of albumin has not been established for every species used in this study, but albumin has been shown to be stable for greater than 3 months in some studies. Freezer storage prior to electrophoretic evaluation is common in clinical and research settings, and when statistically significant effects of freezer storage are reported, they are typically clinically negligible [24, 25, 33, 34]. This suggests that storage prior to electrophoresis should have had minimal effect on the data. If a freezing artifact was present, we expect it affected all samples similarly and therefore would not impact the outcomes of this study. Additionally, while a targeted collection of mammalian samples with ALB < 2.5 g/dL was made, there was not complete matching of the protein concentration in these samples with the protein concentration in the chicken samples, as demonstrated by ANOVA. A difference in the TP or albumin concentration could have impacted the performance of the ALB_BCG_ method or its correlation with ALB_PE_. Notably, there was no detectable difference in A:G_PE_ between groups. This helped to ensure that, if the performance of one of the methods was impacted by the relative concentration of albumin and globulins, disparate A:G between species would not impact interspecies comparisons.

As predicted from published reports, ALB_BCG_ and ALB_PE_ were not comparable in any of the evaluated species. Except for cats near their normal ALB_BCG_ value, chickens showed a similar correlation between ALB_BCG_ and ALB_PE_ when a similarly wide ALB_BCG_ range was evaluated. Part of the higher correlation coefficient in the mammalian data is likely due to a wider range of observed values, as correlation coefficients tend to be higher in datasets with a wider range [35]. The variable degree and direction of bias between species was likely due to the differences in how ALB reacts with BCG in each species, while bovine albumin was used as a reference standard for the assay [20]. Using species‐specific reference standards allows for better performance of the ALB_BCG_ assay, as there appears to be less bias when a species‐specific standard is used; however, having multiple species‐specific albumin assays available in a typical diagnostic laboratory is not tenable [7, 20]. Additionally, the amount of bias may not be clinically relevant in all situations, such as when results are evaluated using a single lab; this is one reason that current international standards minimize the importance of bias when considering analytical performance [36]. The methods were more aligned for cats and ruminants; however, there was still an unacceptably high number of results outside of TEa's limits of agreement.

The comparison of ALB_PE_ and ALB_BCG_ in reptiles was not evaluated in this study, but has been evaluated by others [5, 6, 37]. Both P–B and B–A comparisons in bearded dragons and chelonia demonstrated a similar lack of comparability to that observed in this study for avians and mammals. This might suggest a similar degree of performance for these methods across species.

The results of this study do not explain why some samples had a larger difference between ALB_BCG_ and ALB_PE_; however, it documents the range of differences between the methods. Notably, the greatest difference between ALB_BCG_ and ALB_PE_ was observed in a chicken sample (1.42 g/dL), while the second greatest difference (1.27 g/dL) was in a dog. The statistical evaluation was compatible with a similar average Diff_ABS_ for chickens, dogs, and ruminants. Anecdotal reports of notably divergent results between ALB_BCG_ and ALB_PE_ in other avian species have been published [8]. A similar degree of difference (Pearson's *r* = 0.899 or 0.877, max difference ~1.3 g/dL) was reported when duplicate samples of psittacine plasma were submitted for ALB_PE_ to commercial laboratories [38]. Some of the divergence between colorimetric and electrophoretic methods may be the result of the inaccuracy in the ALB_PE_ method, where two measurements (TP by biuret assay and fraction percent by electrophoresis), each with their own potential for variation, are used to calculate the end result [4]. Method comparison approaches describe the relationship between two methods and cannot, of themselves, determine which method is closer to the true value. This study did not compare the results of spike and recovery experiments with species‐specific albumin between the methods, but that would likely help to confirm the reported use of ALB_PE_ as a reference standard in veterinary species.

Because ALB_BCG_ in this study was performed on a different analyzer than was used for the previously published ALB_BCG_ RIs, and samples were available, we chose to derive new ALB_BCG_ RIs instead of attempting to transfer the previous data [39]. The URL between the two datasets was different (2.4 g/dL vs. 2.1 g/dL); while this could be a result of differences in reference population, it could also reflect the need to update RIs with new equipment.

Ultimately, the data suggest that ALB_BCG_ and ALB_PE_ results should not be considered interchangeable across all evaluated species, as notably divergent results can be observed in many species at an approximately similar frequency. The lack of comparability does not prove that one method is superior to another for diagnostic purposes, but rather that the results from these methods cannot be used interchangeably [28, 40]. Method‐specific interpretation criteria, such as method‐specific RIs, are needed for non‐comparable methods. The clinical utility of method‐specific RIs was modeled coarsely by evaluating interpretive discord between the methods, with most species performing similarly overall. Notably, the two outlying groups were the Avian_Other_ group, where chicken RIs were used to evaluate results from other avian species, and the ruminant group, which used published ALB_PE_ RIs that had not been fully validated for use in our lab. It is likely that species‐specific and laboratory‐generated RIs would have improved the agreement between interpretations for these groups [31]. Nonetheless, this data highlight the importance of using method‐specific RIs for interpretations and suggests that using ALB_BCG_‐specific RIs in chickens may provide clinical utility similar to that seen in dogs, horses, and cats. This should be evaluated in a larger cohort of chickens with a wide variety of clinical conditions before our data can justify the current practice of using ALB_BCG_ to test chicken samples.

This study documented similar analytical and clinical performance of ALB_BCG_ in chickens and other mammals. It does not provide performance‐based criteria for why the method is accepted in some species and rejected in others. It is possible that ALB_BCG_ can perform similarly as a screening method for albumin in all species, or it may need to be replaced by a more updated technique. Further evaluation of the ALB_BCG_ method in all species is warranted.

## Conflicts of Interest

The authors declare no conflicts of interest.

## Supporting information


Table S1–S2.
