# Supplementary material for: Comparison of Electrophoretic and Bromocresol Green Albumin Methods in Chickens and Other Veterinary Species
Source: Vet Clin Pathol. 2025 Jun 2;54(2):171–81. doi: 10.1111/vcp.70015 (PMC12289121; doi:10.1111/vcp.70015)
Supplement: Supplementary file 1 — Table S1–S2. [file VCP-54-171-s001.docx]

**Supplemental Table 1** Z-score and p-value for the comparison of Spearman’s ρ between ALB_BCG_ and ALB_PE ­_using Fisher z-transformation. Comparisons were made between chickens and all data and when the data was partitioned for ALB_BCG_ concentration.

|  | All | | Alb_BCG_ < 2.5 | | >ALB_BCG_ 2.5 | |
| --- | --- | --- | --- | --- | --- | --- |
|  | z-score | p | z-score | p | z-score | p |
| Avian_Other_ | 1.8961 | 0.058 | 1.8961 | 0.058 |  |  |
| Dog | -5.86 | **<0.001** | -0.624 | 0.533 | 0.8256 | 0.409 |
| Cat | -5.417 | **<0.001** | 0.918 | 0.358 | -3.0695 | **0.002** |
| Horse | -2.794 | **0.005** | 0.858 | 0.391 | 1.774 | 0.076 |
| Ruminant | -1.444 | 0.149 | 1.68 | 0.091 | 0.461 | 0.645 |

**Supplemental Table 2** Reference intervals for plasma proteins in chickens measured using colorometric methods.

| **Measurand** | **Units** | **Sex** | **n** | **Mean** | **SD** | **Median** | **Min** | **Max** | **p-value^b^** | **Distribution^b^** | **Method^c^** | **LRL of RI^a^** | **URL of RI^a^** | **CI 90% of LRL** | **CI 90% of URL** |
| --- | --- | --- | --- | --- | --- | --- | --- | --- | --- | --- | --- | --- | --- | --- | --- |
| Albumin_BCG_ | g/dL | All | 131 | 1.71 | 0.36 | 1.7 | 0.8 | 2.5 | 0.058 | N | NP | 0.9 | 2.4 | 0.8-1.1 | 2.3-2.5 |
|  |  | Hen | 103 | 1.75 | 0.36 | 1.8 | 0.8 | 2.5 | 0.006 | NG | NP | 0.9 | 2.4 | 0.8-1.0 | 2.3-2.5 |
|  |  | Rooster | 27 | 1.54 | 0.26 | 1.5 | 1.2 | 2.2 | 0.67 | N | P | 1.0 | 2.1 | 0.9-1.1 | 1.9-2.2 |
| Globulin | g/dL | All | 127 | 3.49 | 0.66 | 3.4 | 2.1 | 5.1 | 0.006 | NG | NP | 2.3 | 5.1 | 2.1-2.5 | 4.8-5.1 |
|  |  | Hen | 99 | 3.56 | 0.69 | 3.5 | 2.1 | 5.1 | 0.137 | N | P | 2.2 | 4.9 | 2.0-2.4 | 4.7-5.1 |
|  |  | Rooster | 27 | 3.20 | 0.41 | 3.1 | 2.4 | 4.1 | 0.387 | N | P | 2.3 | 4.1 | 2.1-2.6 | 3.8-4.3 |
| A:G_BCG_ |  | All | 131 | 0.502 | 0.150 | 0.50 | 0.09 | 0.84 | 0.885 | N | NP | 0.19 | 0.80 | 0.09-0.26 | 0.76-0.84 |
|  |  | Hen | 103 | 0.505 | 0.159 | 0.50 | 0.09 | 0.84 | 0.928 | N | P | 0.19 | 0.82 | 0.15-0.24 | 0.78-0.86 |
|  |  | Rooster | 28 | 0.491 | 0.113 | 0.48 | 0.26 | 0.77 | 0.865 | N | P | 0.26 | 0.73 | 0.19-0.32 | 0.66-0.78 |
| ^b^Anderson- Darling: G, Gaussian; NG, non-Gaussian | | | | | | | | | | | | | | | |
| ^c^P, parametric; NP, nonparametric | | | | | | | | | | | | | | | |
